# Food security definition, measures and advocacy priorities in high-income countries: a Delphi consensus study

**DOI:** 10.1017/S1368980023000915

**Published:** 2023-10

**Authors:** Danielle Gallegos, Sue Booth, Christina Mary Pollard, Mariana Chilton, Sue Kleve

**Affiliations:** 1 School of Exercise and Nutrition Sciences, Queensland University of Technology, Brisbane, Australia; 2 Woolworths Centre for Childhood Nutrition Research, Queensland University of Technology, Brisbane, Australia; 3 College of Medicine and Public Health, Flinders University, Bedford Park, Australia; 4 School of Population Health, Curtin University, Perth, Australia; 5 Enable Institute, Curtin University, Perth, Australia; 6 Dornsife School of Public Health, Drexel University, Philadelphia, PA, USA; 7 Department of Nutrition, Dietetics and Food, Monash University, Clayton, Australia

**Keywords:** Food insecurity, Monitoring, Interventions, Delphi survey

## Abstract

**Objective:**

To establish an international consensus on the definition of food security, measures and advocacy priorities in high-income countries.

**Design:**

A two-round online Delphi survey with closing in March 2020 and December 2021. Consensus was set a priori at 75 %. Qualitative data were synthesised and priorities were ranked.

**Setting:**

High-income countries.

**Participants:**

Household food security experts in academia, government and non-government organisations who had published in the last 5 years.

**Results:**

Up to thirty-two participants from fourteen high-income countries responded to the Delphi with a 25 % response rate in Round 1 and a 38 % response rate in Round 2. Consensus was reached on the technical food security definition and its dimensions. Consensus was not reached on a definition suitable for the general public. All participants agreed that food security monitoring systems provide valuable data for in-country decision-making. Favoured interventions were those that focused on upstream social policy influencing income. Respondents agreed that both national and local community level strategies were required to ameliorate food insecurity, reinforcing the complexity of the problem.

**Conclusions:**

This study furthers the conceptual understanding of the commonly used definition of food security and its constituent dimensions. Strong advocacy is needed to ensure food security monitoring, policy and mitigation strategies are implemented. The consensus on the importance of prioritising actions that address the underlying determinants of household food security by experts in the field from across wealthy nations provides evidence to focus advocacy efforts and generate public debate.

In high-income countries despite agricultural and economic policies that have ensured the availability of enough food, depleted household food security otherwise known as food insecurity (FI) remains an ongoing concern with reports of accelerating use of emergency food relief^(1)^. For example, in 2021 the Trussell Trust in the UK reported a 14 % increase in food assistance compared with the previous year^(2)^. The population prevalence of household FI varies across high-income countries with the most recent government reports ranging from 4% in Australia (in 2011–2012)^(3)^ to 11·2 % in Canada in 2020^(4)^, and 10·2 % in the USA (in 2021)^(5)^. Comparisons are difficult however, as measures are not comparable^(6)^. FI is a complex issue that is not just related to food but, rather, is indicative of material and economic deprivation more broadly. The experience of FI is associated with issues such as the cost of living, un- and under-employment, housing availability and affordability, utilities and energy affordability, discrimination and racism, poor health and well-being as well as structural and interpersonal violence^(8,9)^. The consequences of persistent FI can be detrimental to mental and physical well-being across the life course including poor child growth and development, and in children and adults, malnutrition, overweight and obesity, non-communicable diseases and mental health issues^(9–11)^.

The USA, Canada, New Zealand, Australia, member countries of the European Union and the UK are considered industrialised and high-income countries with comparable (although different) agricultural policies^(12)^. They each have different health systems and social protections. Some countries rely on publicly funded food assistance programmes, for example, the USA, but increasingly utilise charitable food relief to supplement food assistance shortcomings^(13)^. Globally, discussions on strategies to ameliorate household food and nutrition security have resulted in a myriad of approaches, reflecting the complexity of the issue^(14)^. The United Nations Food and Agricultural Organisation (FAO) attempts to measure the political commitment to and capacity for addressing FI in low-income countries^(15)^ and recommends countries use the Food Insecurity Experience Scale to enable comparisons^(16)^. There is, however, limited dialogue regarding the most appropriate responses within high-income countries.

An effective policy response requires a clear and shared definition of a problem^(17)^; the most cited definition of food security is the FAO’s consensus definition published in 1974 and refined and restated in 2012^(18)^. Specifically, food security exists when *‘all people at all times have physical, social and economic access to food, which is safe and consumed in sufficient quantity and quality to meet their dietary needs and food preferences … allowing for a healthy and active life’* (p. 8)^(18)^. When this does not occur a state of FI exists. This definition moved away from a sole focus on malnutrition, minimum caloric intakes and preventing starvation to increasing attention on diet quality, health promotion and protection against diet-related chronic disease. This shift signals an understanding that food security is more than the absence of malnutrition but instead reflects a complex intersection of economic, social, cultural and biological factors that influence the quality and quantity of food available and accessible to households^(19)^. Concomitantly, the measurement of food security at a population level has moved from proportions of the population who are undernourished towards food security experience scales at the household and individual level^(20)^. FI experience scales contribute to evaluating progress towards the Sustainable Development Goals, including the goal for zero hunger as well as those goals that contribute to food security (e.g. poverty alleviation, good health and well-being, education, clean water, affordable energy, life on land, peace and justice).^(16)^ For operationalisation, this definition requires a complex matrix of dimensions to be in place to achieve food security including food: availability; accessibility; utilisation; stability, agency and sustainability^(21)^. The conceptualisation and determinants of each dimension and their interactions are contextually dependent, and how their absence leads to FI is complex.

Candel described food security as a ‘wicked problem’ that is ‘ill-defined, ambiguous contested and highly resistant to solutions’ (p. 288)^(22)^. In high-income countries, FI in all its manifestations of human experience (from being worried about the ability to put food on the table to going hungry for a day or more than a week) is relatively hidden. Population-level FI prevalence is relatively low, and those at the severe end of the scale are in the minority; however, the inability to consume a diet conducive for an active and healthy life due to FI is more common^(3–5)^. Only two high-income countries (USA and Canada) regularly monitor FI at the household level using robust instruments, and a response led across the whole of government and engaging civil society action is rare, Brazil being the only example^(23)^.

How you define a problem and measure it influences the policy response^(17)^, and if you do not measure it, it remains invisible providing no impetus for the generation of solutions^(24)^. A clear problem definition is needed in order to assess the portfolio of interventions available to address it, in this case, to tailor a fit-for-purpose policy response to FI in a specific area^(1)^. The process involves four stages: first, defining the policy problem; second, considering what could or should be done; third, deciding options and finally, monitoring implementation and impact. There is reportedly confusion regarding the definition and understanding of food security and therefore FI, in part to do with the context in which it is considered (national, community, household or individual level^(25)^) and a lack of understanding or agreement on the drivers and consequences of the problem. To generate effective responses, there needs to be agreement on a definition of food security (and as an extension its absence – FI) and to understand the determinants. A clear definition provides the context for action and assists with identifying the desired outcomes. Definitional precision is an important first step in the intervention selection process^(24)^ and in holding governments accountable to using policy levers to address the underlying determinants to ensure food security in a meaningful and sustainable way^(26)^.

A series of policy solutions to address food and nutrition problems have been recommended over the years^(19)^. However, due to the complexity of the issue of FI and the nature of the problem in different political and geographic contexts, and the fact that the solutions often lie across sectors, identifying the ‘right’ policy options has been difficult. Selecting from a portfolio of interventions, from social and public policy and legislation to nutrition education, can be difficult. There are food policy packages to improve diet and prevent diet-related non-communicable diseases, such as the World Cancer Research Fund International’s NOURISHING framework^(27)^ which are continuously updated to build momentum for policy implementation^(28)^, but there is little international consensus on the options to address FI in high-income countries.

The aim of the Delphi study was to establish an international consensus on the definition of food security and strategies for surveillance and to identify and prioritise key actions to address household FI in high-income countries.

## Methods

An online Delphi series methodology was used to survey international experts in household food security from academia, government and non-government organisations via email. The Delphi method is used to ‘obtain the most reliable consensus of opinion of a group of experts’ (p. 458)^(29)^. It is a structured, iterative, cost-effective approach to collecting opinions, polling for feedback and making group-based judgements on complex issues from a larger panel, in a systematised way that is anonymous^(30)^. The method has been used extensively within health and food systems research to develop indicators and frameworks including, for example, in food system sustainability^(31)^, food risk governance^(32)^ and measurement development^(33)^. The Delphi was the method of choice as it allowed access to a broad expert group that was geographically dispersed and enabled qualitative analysis combined with consensus building on a complex issue^(30)^. This made it preferable to other methods to gain consensus such as consensus development panels or nominal group techniques which require face-to-face groups^(34)^. The Delphi series was used to gain consensus on definitions and measures and to prioritise actions to address FI^(30)^. In this current study, Round 1 of the modified Delphi consisted of open-ended questions to enable exploration of the key areas and Round 2 presented these results for consensus and ranking.

### Sample

The purposeful sampling method was chosen selecting participants working across a range of relevant areas (academia, public sector or individuals from government agencies or non-government organisations who had published peer-reviewed or publicly available reports on household FI). All participants resided in high-income countries as defined by the World Bank (2019)^(12)^. Delphi panels are often considered valid if they consist of between 15 and 60 participants^(35)^; however, it is recognised that for some issues there are a relatively small number of recognised experts and in these cases it is imperative that the knowledge and opinions of these experts guide best practice^(30)^. However, we do acknowledge that this Delphi includes only those with learned expertise and ignores those with lived experience whose contribution would strengthen the results^(36)^.

### Instrument design and data collection

For Round 1 of the Delphi series, the authors reviewed the literature and used the FAO Food Security and Commitment and Capacity Profile Methodology Paper^(15)^ to develop a series of open-ended questions to assess the objectives. The survey which incorporated thirteen questions was field-tested by colleagues and refined based on feedback. The questions outlined a definition of FI and asked about definitions used in each participants’ country and for a user-friendly definition that could be used with the general public; tools used to measure household FI; how often population measures were conducted; opinions on preferred measurement tools; the primary determinants of household FI; government and community/not-for-profit interventions to alleviate FI; and to gather information about the evaluation and effectiveness of interventions implemented. Collected demographic information included the country of residence and employment, qualifications and expertise, length of time working in the area of household food security and the type of organisation they currently worked for. See online Supplementary file 1 for a copy of the survey.

### Round 1: Dissemination

One hundred and forty-nine participants were identified and contacted via email and invited to complete the online survey deployed using Qualtrics^®^ between October 2019 and March 2020. The survey took approximately 40 min to complete. A reminder email was sent 2 weeks later, but due to the number of participants sending ‘out of office’ responses the survey was kept open, and reminders sent again on their stated return to office date. All participants were also asked to complete a conflict of interest statement.

### Round 2: Survey instrument development

The authors revised the questionnaire based on the feedback from Round 1 and in Round 2 of the Delphi series, participants were asked to indicate their level of agreement with definitions and dimension and domain summary statements and to prioritise strategies and actions. A six-point Likert scale (from strongly agree to strongly disagree with a do not know response) commonly used in Delphi surveys was used to measure consensus statements with the opportunity for qualitative amendments to definitions and statements provided^(30)^. Consensus was set prior to the first round and taken as equal to or above 75 % strongly agree and agree^(37)^.

The definition of food security included each dimension of food security: (i) availability; (ii) access (economic, social and physical); (iii) utilisation (access to household equipment, food literacy, time, water, sanitation and hygiene (WaSH)) and (iv) stability. Just after the completion of Round 1 of the series, the FAO High Level Panel of Experts released a report that included an update to their definition of FI with additional dimensions of ‘agency’ and ‘sustainability’. These dimensions were included in Round 2 of the Delphi series which sought to reach agreement on a preferred FI measurement tool, essential components and considerations for implementation. See online Supplementary materials 2. In Round 1, participants identified that the definition of food security/insecurity was not well understood by members of the public. The definition was described as using high level language and difficult concepts. Consequently, in Round 2 definitions suitable for the general public based on Round 1 suggestions were presented to the panel for prioritising (data not presented). All definitions were analysed for readability (https://www.webfx.com/tools/read-able/). This tool provides an age range to indicate the level of cognitive understanding targeted. The aim is for upper primary school age, that is, about 11–12 years of age. Participants ranked definitions according to preference with one the most preferred and five the least preferred. Rank scores were informed by the method outlined by the James Lind Alliance^(38)^. For these definitions, rank scores were calculated by adding reverse scores 1 = 5, 2 = 4 3 = 3, 4 = 2, 5 = 1 and dividing by the number of participants. The higher the number, the higher the preference.

All intervention strategies proposed by participants to address food security in Round 1 were presented for prioritisation in Round 2. Interventions were grouped into three categories: (i) national level primary policy and social protections (*n* 17, e.g. social welfare payments, universal basic income) – these addressed the root determinants of FI; (ii) national strategies (*n* 9, e.g. universal free school meals) these were nationwide food strategies; and (iii) community or local level strategies (*n* 14, e.g. social supermarkets, cooking programmes) – including place-based, grassroots interventions. Participants ranked their three highest priority strategies in each category, with one being the highest and comments were also invited.

### Round 2: dissemination

All participants who responded to Round 1 were invited to Round 2 in October 2021. The timing of Round 2 was delayed due to the COVID-19 pandemic and stayed open until December 2021 to ensure consistency with Round 1. All participants received one reminder and if an ‘out-of-office’ response was received a reminder was sent upon their return. Two rounds were considered adequate as a tool of analysis as clear patterns emerged after the second round, negating the need for additional rounds^(30)^.

### Data analysis

A pragmatic qualitative description analysis approach was used to investigate the open text responses from Round 1 conducted in three distinct phases commonly used to theme data. All authors were involved in the three phases: (1) data familiarisation, listing recurrent ideas and issues; (2) open-coding and (3) group discussion to compare codes and synthesise (categorising) for agreement on data representation in Round 2 (including wording and response options for each question)^(39)^.

Following Round 2, the data were extracted and the same process outlined above was followed to discern patterns for any wording changes which were then cross-checked by all authors to ensure accurate representation. Verbatim quotes were used as exemplars of points made. As participants prioritised different strategies, items were reverse scored according to their preference (e.g. 1 = 20 points, 2 = 10 points and 3 = 5 points). This enabled strategies to be ranked with top ranking gaining maximum points^(38)^.

## Results

### Participants’ characteristics

The Round 1 survey was sent successfully to 130 participants across seventeen different countries including Australia, Canada, Denmark, England, Finland, France, Italy, Northern Ireland, the Netherlands, New Zealand, Norway, Portugal, Scotland, Spain, USA and Wales. There were forty-three responses received (seven were opened but did not consent to participate, four consented but did not provide any responses) resulting in thirty-two participants and a 25 % response rate.

Round 2 was sent to twenty-one respondents who agreed to be recontacted (email delivery failed for four respondents and alternative contact addresses could not be found); nine responses were received with eight useable surveys representing a 38 % response rate. Table 1 provides data on respondents’ geographical location and work area/expertise.

**Table 1:** Geographical location and discipline areas of respondents

	Round 1		Round 2	
Geographical location				
Country	*n*	%	*n*	%
Australia	12	38	3	38
Canada	2	6		
Denmark	1	3		
England	1	3	1	12·5
Finland	1	3		
France	1	3		
Germany	1	3		
Ireland			1	12·5*
Netherlands	1	3		
New Zealand	1	3		
Northern Ireland	1	3	1	12·5
Scotland	2	6		
Spain	1	3	1	12·5
USA	7	22	1	12·5
Total	32	100	8	100
Work area/expertise†	Round 1		Round 2	
Nutrition	16	36	5	50
Public health	15	33	3	30
Sociology/Anthropology	5	11	1	10
Epidemiology/Statistics	6	13	1	10
Social Justice/Food sustainability	2	4		
Consumer Research	1	2		
Years of experience in food security				
< 5 years	5	15·6	0	0
5–10 years	5	15·6	2	25
11–20 years	6	18·8	3	37·5
> 20 years	16	50	3	37·5

* One respondent indicated Ireland in Round 2 and not in Round 1.

† More than one response per respondent allowed.

### Definition of food security

Most definitions of FI identified by responders were based on those described in the peer-reviewed literature^(5,18,26,40)^ but the final definition was extended to incorporate several concepts that were perceived to be missing. The final food security definition presented was based on the recommendations from respondents (see Box 1) and had 100 % agreement, with suggestions to incorporate ecological sustainability, affordable food and a terminology change from ‘emergency’ to ‘charitable’ food relief.

Box 1 Definition of food security

Food security exists when all people, at all times, have regular and reliable physical, social and economic access to sufficient safe, nutritious and culturally relevant food that meets their dietary needs and food preferences. This is supported by an environment of ecological sustainability, adequate sanitation, health services and care for an active and healthy life. This includes the assured ability to acquire acceptable, affordable foods in socially acceptable ways without resorting to charitable food supplies scavenging, stealing and other coping strategies.

The following quote captures respondents’ sentiments regarding the definition *‘I think the above definition of food security is useful in national and community sense [that is] good to use for national policy’* and *‘I really appreciate the inclusion of the culturally relevant aspect’.*

Participants (*n* 10) ranked the definitions for the general public with the two most preferred definitions presented in Box 2.

Box 2 Preferred general public definitions of food insecurity

Food insecurity is the uncertainty about the ability to obtain food. It means you have to settle for less food or food of low quality for your family (readability score suitable for 14–15 year olds)

Food insecurity is the inability of people to access, adequate, affordable and acceptable food (readability score suitable for 21–22 year olds)

### Food security dimensions and domains

Table 2 summarises the food security dimensions, their definitions and levels of agreement.

**Table 2:** Levels of agreement with the dimensions of food security definitions^†^

Dimension	Definition	Level of agreement* %
Availability	Healthy nutritious foods *free from adverse substances* need to be available at the national, community and local level. Sufficient food variety needs to be available to enable individuals to choose foods that meet their physical, social and cultural needs	71
Access	To be divided into three domains: physical, economic and social The one neutral comment indicated: *I think the three criteria should be physically available, economically affordable and convenient to obtain’* (USA)	89
Physical	Food needs to be accessible where people live. Individuals, households and communities should have access to infrastructure and strategies that optimises access to food without undue burden on household resources	75
Economic	Individuals, households and communities need sufficient income to meet the needs of daily living without stress, including housing, *healthcare, childcare, education, transport,* water, utilities, communication and food. Healthy food should be affordable *to all* *	89
Social	All members of communities have access to food irrespective of their circumstances and *personal characteristics (such as* gender or age*)*. Food can be acquired in ways that are socially acceptable and just*	88
Utilisation	Divide into four sub-domains: access to household equipment, food literacy, time and water, sanitation and hygiene (WaSH). These are all required collectively for food security to be achieved	75
Access to household equipment	Individuals and households require access to *spaces* and equipment that are *safe and* in good working order that enables them to *store and prepare food* that meets their health, social and cultural needs*	86
Food literacy	Communities, households and individuals need the knowledge and skills that empower them to plan, manage, select, prepare and eat foods that meet their health, social and cultural needs	75
Time	Time pressures/demands of individuals or households that impact on food provisioning (e.g. to *obtain,* plan, manage, select and prepare food). The relationship between the time available (work, parenting and other demands) *v*. the *time necessary* to undertake food provisioning tasks*	75
Water, sanitation, hygiene	All communities, households and individuals need access to safe and affordable water supply and sanitation systems that prevent disease and the knowledge and skills to keep the water and food supply safe to consume	75
Stability	All communities, households and individuals can ensure the availability of, access to and utilisation of nutritious food in the event of shocks that may occur at a global, national, community or household level. This would include the effects climate change, economic crises, pandemics, violence (conflict as well as personal violence and trauma) and cyclical events (e.g. weather)	88
Agency	Individuals or groups having the capacity to act independently to make choices about what they eat, the foods they produce, how that food is produced, processed and distributed, and to engage in policy processes that shape food systems. The protection of agency requires socio-political systems that uphold governance structures that enable the achievement of Food and Nutrition Security for all‡	88
Sustainability	Food system practices that contribute to long-term regeneration of natural, social and economic systems, ensuring the food needs of the present generations are met without compromising the food needs of future generations‡	88

* The italicised words are additions made from the comments from participants. It should be noted these have not been represented to participants for further agreement.

† Agree and strongly.

‡ These dimensions were published after Round 1 by FAO (2020) and included to confirm their addition as dimensions in Round 2.

### Measurement of household food security

Box 3 shows the features of household food security measurement where consensus was achieved, with 86–100 % agreement. All participants agreed with regular (annual) population monitoring and surveillance of household food security in high-income countries and the USDA Food Security Survey Module (USDA FSSM) (six, ten and eighteen question versions) as their preferred tool.

Box 3 Consensus on measures of household food security

**Table:** 

Instrument must be able to:	Agree (%)
Assess severity	100
Capture worry about running out of food	100
Detect hunger	100
Allow comparison across time-points	100
Be applied at the household and the individual level	100
Be administered in a variety of forms (interviewer, self-complete)	100
Ensure able to be administered for all people experiencing food insecurity are captured (such as no telephone, living in caravan/trailer parks, no fixed address)	86
Be used over different time periods (such as over last 12 and 3 months, last month)	86
Identify the impact on children	86

### Intervention priorities

Participants ranked their top three intervention priorities from a comprehensive list of potential interventions grouped according to the socio-ecological model (e.g. operationalised at a policy, community and individual level). Tables 3–5 summarise the ranked intervention priorities for policy, national and community strategies, respectively. Quotes are provided to highlight the responses. Some participants commented that they ranked national and community strategies only because they were required to, but the focus should be on policy.

**Table 3:** Ranking top three intervention priorities for policy nationally

Policy priorities	Rank score
Income: social welfare payments (social security benefits, unemployment benefits, single parent support, housing assistance); universal basic income; legislated minimum wage – minimum wage is a living wage (access)	50
Comprehensive poverty alleviation strategy (access)	25
Address under- and insecure-employment and working conditions (employment and working conditions) (access)	20
Multi-sector food and nutrition security policy (availability, access, utilisation)	15
Livelihood and employment generation and promotion for growth/scalability (employment and working conditions) (access)	10
Measure, map and report food insecurity (availability, access)	10
Government funded food security programmes, for example, SNAP, WIC (access)	5

SNAP, Supplemental Nutrition Assistance Program; WIC, Women, Infants and Children.

Comments regarding these interventions included ‘Address the structural causes of food insecurity with policy solutions to reduce the gap between the rising cost of living and income through a ‘cash first approach’ either through real living wages or benefit maximization via a fit for purpose social security system.’ (Northern Ireland), ‘The top variable in food insecurity is jobs and wages’ (US) and ‘We need to address the root causes of food insecurity – income, poverty and then we need an overarching nutrition policy to tie it all together and plan for a future with nutrition and food security for all’ (Australia).

**Table 4:** Ranking top three intervention priorities for national strategies

National strategies to alleviate food insecurity with a food focus	Rank score
Subsidies on healthy food choices (access)	50
Map and evaluate current national and local programmes (availability, access)	40
Universal free school meals (e.g. breakfast, lunch, snacks) (availability, access)	40
Improvements resilience and agility of food supply chains and environments (availability)	30
Pricing strategies in the retail food environment (access)	25
Taxes on unhealthy food choices (access)	20
Food literacy programmes in schools (utilisation)	10
Means-tested school meals (access)	5

Comments included: ‘We need to start with young children to ensure they are food secure to educationally attain and become economically active in the future and we need to incentivise healthy choices at the population level to reduce potential for future public health crises (put universal free school meals as #1)’ (Northern Ireland) and ‘…education alone cannot change the situation, nor fiscal incentives as they are subject to people’s choices and priorities. Therefore, I would support structural changes along the food system. I still think that food literacy is essential, but not only at the school level’ (Spain) and finally, a caution that ‘too many programmes are left without robust evaluation’ (England).

**Table 5:** Ranking top three intervention priorities for community or local level strategies

Community-local level strategies	Rank score
Community suite of services (e.g. food access, financial access, social inclusion, growing food, provides food relief, financial and housing advice, counselling, links to training and education, employment opportunities, etc.) (availability, access, utilisation)	70
Food hubs (availability, access)	40
Social supermarkets or social solidarity stores (access)	35
Emergency food relief, food assistance programmes (access)	20
Cooking or other food literacy programmes (utilisation)	20
Social cafes (access)	15
Budgeting programmes (access)	10
Food waste reduction campaign (utilisation)	10
Farmers markets and community food markets (availability, access)	10
Meal provisioning for community dwelling elderly, for example, Meals on Wheels, congregate (meals provided for the elderly at a venue) (access)	10
Community kitchens (utilisation)	5

Additional comments emphasised the need to reconnect people to local food systems to *‘make healthy food choices easily accessible, affordable and available’* (Northern Ireland), and a criticism that the options *‘missed the point that people need more money to be able to afford food and that housing, transportation, childcare, and health care need to be free or more affordable.’* (USA) and *‘Again, many of those interventions are insulting to low-income people. Food literacy and person ‘budgeting’ aren’t the main problems – low wages and high housing costs are the main problems.’* (USA) suggesting that the options in the national policy level were preferred and the priority. There was an acknowledgement that *‘many community interventions are not shown to be effective for FI. Hard to evaluate.’* (Australia).

## Discussion

The international community via the mechanisms of the United Nations has, over time, sought consensus on the right to food and to define food security for multiple contexts. However, this is the first time that an international consensus has sought to contextualise and operationalise the definition of food security and its dimensions for high-income countries, where severe FI is less prevalent. This refined definition will support prioritisation of strategies to address the problem and to identify key areas for action. A consensus was reached on the definition of food security and its components, and how it should be monitored. Household material deprivation and poverty in high-income countries were recognised as the underlying determinant of FI by participants in this Delphi series, and interventions to address these determinants were prioritised. FI was also recognised as an outcome of the matrix of structural violence that perpetuates systemic disadvantage.

This Delphi survey achieved consensus, from a panel of thirty-two (Round 1) and eight (Round 2) international experts from thirteen high-income countries who unanimously agreed on the definition of food security, five of six dimensions and all sub-domains (75–89 %). Consensus was not reached on the definition of the ‘availability’ domain (71 %) of food security, due mainly to concerns around the inclusion of *‘free from adverse substances’* in the statement which was considered to be vague and needed to be defined. The USDA FSSM tools were unanimously rated as the preferred measurement instruments and there was agreement on all monitoring elements. National policy interventions to address the underlying determinants of FI were considered the most important approach to be prioritised^(8,41)^.

### Definition and dimensions

Public health concepts require precise definitions that contribute to conceptual clarity that in turn inform identifying the underlying mechanisms that need to be investigated and dismantled. As social and cultural contexts shift, as the science evolves and as our understanding of the concepts becomes more sophisticated, definitions need to be updated^(42)^. Clarifying the definition of the overall concept (in this case food security) as well as the individual dimensions provides the opportunity to develop a more robust and defensible consensus that will assist with identifying key actions across sectors that are mutually beneficial^(43)^. This provides a clearer remit for policy makers and practitioners to develop in-country solutions and evaluate them appropriately to assess progress towards the Sustainable Development Goals^(44)^.

The agreed definition of food security reduces the ambiguity in previous definitions^(45)^. There are important additions, including the overt reference to sustainability, cultural acceptability and procuring foods in ways that are socially acceptable. The definition highlights FI is not just a product of emergency situations or ‘shocks’ but a chronic condition in high-income countries. As such, the reliance on charitable food relief and in the USA on government-funded initiatives such as the Supplemental Nutrition Assistance Program and Women, Infants and Children both of which have strict eligibility criteria as the primary answer to household FI, as a sustainable long-term response, is inadequate. Additionally, the international consensus response deems charity specifically as socially unacceptable^(46,47)^.

The emphasis on the availability and access to a diverse food supply with high nutritional quality and affordable food in the definition dimensions also points to potential policy interventions. Clarifying the utilisation dimension with the addition of ‘timeliness’ and food literacy is also important as there is growing acceptance of time as a determinant of health recognising the significant cognitive load required to put food on the table when accessibility and availability are disrupted^(48)^. There was a strong concern that a focus on these elements potentially puts the onus of responsibility on the individual. For example, the food literacy sub-domain, such that government may continue to focus on building personal skills at the expense of addressing the underlying structural determinants (such as lack of income, affordable housing). Food literacy can only be enacted if the food is available and accessible in the first instance and may extend the time that a household or individual can feed themselves but is useless when food is no longer available or when money runs out^(49)^. There was consensus regarding the additions of agency and sustainability to the dimensions^(50)^.

### Measuring food insecurity

A comprehensive definition can assist monitoring and inform the development of strategies and evaluation of actions. There was consensus that there needs to be regular and reliable monitoring of food security and therefore its absence (FI). Irrespective of the tool used, the severity of the FI experience in both adults and children needs to be captured. Comparison across time-points and between countries was considered essential. The FAO Food Insecurity Experience Scale, an 8-item survey based on the USDA HFSSM, is currently being used to compare FI prevalence between countries^(51)^; however, within country monitoring is still needed to inform national and local strategies. Surveillance systems should monitor population prevalence and specific sub-population groups at risk of FI. The current situation where governments in many high-income countries do not monitor FI has led to some civil society organisations taking on the responsibility of collected and reporting on food security. These organisations have a vested interest in potentially overstating the extent of the problem to ensure ongoing government and philanthropic funding. Despite their best efforts, these civil society stakeholders do not have the funding or infrastructure to conduct or interpret population representative surveys, and the onus of responsibility should not be with them to undertake such monitoring.

The participants agreed the USDA HFSSM is the preferred tool for the measurement of household food security. However, this tool and the Food Insecurity Experience Scale only measure financial access and not the other aspects in the outlined dimensions^(52)^. Based on this current research, it is recommended that additional measures beyond those that are income based would be useful. This could include the deployment of the USDA HFSSM as the core module to identify financial FI but then linking to other measures or incorporating this measure into composite indexes. Such comprehensive, ongoing data would inform the equitable distribution of resources and enhance the development and evaluation of policy and strategies that enhance agency and sustainability.

Despite the recognised value of comprehensive nutrition monitoring and surveillance systems for policy purposes, few high-income countries have routine systems. The yearly National Health and Nutrition Examination Survey in the USA is the exception^(53)^, with other countries monitoring nutrition on average every 8–10 years. Most countries comprehensively monitor foodborne illness due to the immediate and devastating impacts of outbreaks; however, comprehensive food security monitoring systems are lacking. Food price monitoring is also limited; FAO monitors commodity prices at an international level^(54)^ but local data relevant to the definition and experience of FI are not. International Standardised Affordability and Pricing (ASAP) protocols are being recommended to compare food affordability but are still in development and difficult to administer due to the commercially sensitive nature of food pricing data. Collecting this information, coupled with information on income and labour dynamics, would provide cross-portfolio intelligence to inform effective policy^(55)^.

Given the elements that needed to be captured, the resulting definition for food security was long and complex, and further research is needed to assess the suitability of its use in practice. There was also a recognition among participants that the definition of FI was not suitable for the general public and that this contributed to the lack of recognition of the problem. Attempts to define a version of the definition that was more easily understood were inconclusive and would need to be developed further, with the potential use, target audience and intent of the definition clear prior to its. We were unable to develop wording that could be understood at an upper primary school level of education. This highlights the need to work with those with lived experience of FI to refine the definition so that it is understood by the entire population.

### Interventions to address food insecurity

The main finding of this study was the need for a comprehensive suite of interventions to address FI from policy to community level responses, with priority given to addressing the key economic determinants of FI. There was a clear and overwhelming response that FI cannot be fixed by providing food^(56)^. Rather, action on the upstream structural barriers that limit access to a universal basic income, living wages or social supports (e.g. support during unemployment, illness, disability) is required. This is not unexpected and aligns with the Food and Agriculture Dialogue on Food Security which identified making ‘healthy diets affordable and accessible through social protection (that is, cash transfer programmes) and income generation policies’ (p. 4)^(14)^. A livelihoods approach would indicate there has been a failure of both entitlements (access to health services, safety, income, housing and education) and the development of capabilities – which are an outcome of entitlements (such as literacy, reasoning and judgement and the ability to work) to ensure households are able to put food on the table^(57)^. Government policy should address salient needs of individuals and households including housing, meaningful, stable and secure employment opportunities, transport, as well as affordable health and childcare.

Participants ranked strategies that would need to be supported nationally with a food focus, more highly if they were universal with minimal eligibility criteria. Inequitable and stigmatising strategies can negatively impact both physical and mental health creating a cycle of impoverishment^(58)^. Some strategies such as food subsidies and universal school meals are costly and highly political or involve the integration of currently siloed systems (e.g. education, health, agriculture). Cultural context, feasibility, acceptability and political will are considerations when assessing the appropriateness of interventions. The current consensus provides a starting point for the types of interventions that may guide policy and political conversations and generate public debate^(59)^.

At the local level, there was clear agreement that a suite of services that go beyond the provision of food was preferred, however, although these will add value there is no expectation that they will resolve FI^(56,60)^. Community level interventions need robust evaluation focusing on the outcome of the immediate alleviation of hunger through the provision of food, rather than their likely impact on FI, for example, through increases in financial literacy. Examples of interventions with robust evaluation include the Growing Communities initiative in London and Kitchen Table Conversations in Cardinia, Victoria, Australia, and the ‘STOP’ Community Food Centres in Canada^(61–63)^

### Strengths and limitations

This Delphi of high-income countries brought together experts with learned experience from different discipline areas across thirteen different countries all with varying approaches to maintaining food security. However, it does have limitations. The contexts in low- and middle-income countries vary from those in high-income contexts and the definitions and consensus of priorities and approaches could be markedly different. Consequently, the consensus outlined here cannot be generalised until verified across contexts. The response rate in Round 2 was lower than expected; however, there is acknowledgement in public health consensus that it is the quality of the responses rather than the quantity that is necessary. The low numbers in Round 2 may have been due in part to the extended period of time between Rounds 1 and 2 due to COVID-related delays. Finally, this Delphi privileges the views of those with learned expertise and fails to include those with lived experience. Additional work with those who have or who are experiencing FI is necessary to ensure concepts and approaches resonate with all.

## Conclusion

To our knowledge, this is the first attempt to develop a consensus definition and priorities for action for maintaining household food security in high-income country contexts, among scholars, and advocates outside of the UN structure. This consensus will assist in directing measurement of household food security, focusing on priorities for policy development, and an advocacy platform to advance actions to ameliorate household FI in high-income countries. The definition provides conceptual clarity of the overarching definition and constituent dimensions. We strongly urge researchers, public servants and non-government organisations to use this definition to inform advocacy, research, strategy development and evaluation frameworks. The Delphi process confirmed the importance of responses that go beyond charitable food provision and provides evidence for focusing advocacy resources on stimulating public debate. The results here are not meant to be prescriptive but rather seek to enhance discussion on ways to build momentum to progress tangible and sustainable actions to ensure equitable food security for all in high-income countries.

## Supplementary material

For supplementary material accompanying this paper visit https://doi.org/10.1017/S1368980023000915. click here to view supplementary material
