# Supplementary material for: Food security definition, measures and advocacy priorities in high-income countries: a Delphi consensus study
Source: Public Health Nutr. 2023 May 5;26(10):1986–96. doi: 10.1017/S1368980023000915 (PMC10564592; doi:10.1017/S1368980023000915)
Supplement: Supplementary file 1 [file S1368980023000915sup001.docx]

Household food insecurity in high income countries: Developing a consensus

Start of Block: Default Question Block

**Description**

Household food insecurity is the ability of households to physical, social and economic access sufficient affordable, quality food for an active and healthy life. In many industrialised countries such as Australia, New Zealand, Canada, the United Kingdom and the United States of America the focus is on the ability to continue to produce food in the face of climate change. This focus is vital to ensure the availability of adequate food nationally however little attention is given to the distribution of that food to households. At a population level over one in ten households are food insecure and in low to middle income households this can increase to at least one in four. This research aims to develop an international consensus in on the definition, framing, monitoring and responses to food insecurity in high income countries.   The survey is designed to assist in the development of a consensus regarding the issues around the political and programmatic agendas for household food insecurity in high income countries. While many of these agendas will be localised to the within-country context, there are broader aspects for which an international perspective will be valuable.   You are invited to participate in this research project because you have been actively involved in researching, evaluating or developing programs in household food insecurity in either Australia, Canada, New Zealand, UK or the USA.

**Participation**

The survey is primarily qualitative and depending on the depth of your responses will take between 30 and 45 minutes to complete, online.   There are some preliminary demographic questions based on your role with respect to household food security and in the interests of transparency there is also a conflict of interest statement to complete.

There are 13 open-ended questions asking you about household food insecurity. For example:

·         What are the primary determinants of household food security insecurity in your country? ·         Do you think there is political will to improve household food security in your country?

Responses from this survey will be collated and themed firstly for individual countries (depending on the number of responses) and then across countries. These themes will be re-presented in the form of a Delphi at a later stage in order to achieve consensus. No individual responses will be identifiable but verbatim quotes (identified at the country level) may be used in reporting, presentations or peer-reviewed papers.

Your participation in this research project is entirely voluntary. If you agree to participate you do not have to complete any question(s) you are uncomfortable answering. Your decision to participate or not participate will in no way impact upon your current or future relationship with QUT, Drexel, Curtin, Flinders or Monash Universities. If you do agree to participate you cannot withdraw from the research project after submitting your responses as your responses are not-identifiable.

You will be able to review your responses before submitting and save a copy of your responses after submitting the survey.

If you would like to participate in the further rounds of the Delphi process we will ask for your name and contact details at the end of the survey. This information will be kept separately to your responses.

**Expected benefits**

It is expected that this research project will not directly benefit you. However, it may benefit the development of national and international agendas for the alleviation of household food insecurity. You will be able to use the results from this process for your own organizational advocacy.

**Risks**

There are no risks beyond normal day-to-day living associated with your participation in this research project.

**Privacy and Confidentiality**

All comments and responses are anonymous and will be treated confidentially unless required by law, or regulatory or monitoring bodies, such as the ethics committee.  The names of individual persons are not required in any of the responses.   Any data collected as part of this research project will be stored securely as per QUT’s Management of research data policy. Please note that non-identifiable data from this research project may be used as comparative data in future projects or stored on an open access database for secondary analysis.

This survey has ethics clearance from the Queensland University of Technology Human Research Ethics Committee Approval Number 1800001126

**Research Team**

Professor Danielle Gallegos  Queensland University of Technology [danielle.gallegos@qut.edu.au](mailto:danielle.gallegos@qut.edu.au)

Professor Mariana Chilton      Drexel University

[mmc33@drexel.edu](mailto:mmc33@drexel.edu)

Dr Sue Booth                          Flinders University

[sue.booth@flinders.edu.au](mailto:sue.booth@flinders.edu.au)

Dr Sue Kleve                          Monash University

[suzanne.kleve@monash.edu.au](mailto:suzanne.kleve@monash.edu.au)

Dr Christina Pollard                   Curtin University

[C.Pollard@curtin.edu.au](mailto:C.Pollard@curtin.edu.au)

Please do not hesitate to contact any of the research team members if you have questions.

**Consent to Participate**

Answering yes to the consent question means that you consent to participate and your responses even if you only partially complete the survey will be used.  

End of Block: Default Question Block

Start of Block: Block 5

I consent to participate

- Yes (4)
- No (5)

End of Block: Block 5

Start of Block: Block 6

Thank you for considering this survey for participation please answer two quick questions

Q1 In which country do you mainly reside and work?

- Australia (1)
- Canada (2)
- England (3)
- New Zealand (4)
- Ireland (5)
- Northern Ireland (6)
- Scotland (7)
- United States of America (8)
- Wales (9)
- Other: please specify (10) ________________________________________________

Q2 Please state the reason you were unable to participate

- Lack of time (1)
- No longer in the area of food insecurity (2)
- I am not authorized to respond in my current work capacity (3)
- Not interested (4)
- Other (please state) (5) ________________________________________________

End of Block: Block 6

Start of Block: Demographics

In which country do you mainly reside and work?

- Australia (1)
- Canada (2)
- England (3)
- New Zealand (4)
- Ireland (5)
- Northern Ireland (6)
- Scotland (7)
- United States of America (8)
- Wales (9)
- Other: please specify (10) ________________________________________________

Please select up to two responses that most represent your qualifications/expertise

- Anthropology (1)
- Epidemiology (2)
- Global studies/international development (3)
- Medicine (4)
- Nutrition (5)
- Paediatrics (6)
- Public health (7)
- Sociology (8)
- Other: please specify (9) ________________________________________________

How long have you been working in the area of food security?

- (1)
- 5-10 years (2)
- 10-20 years (3)
- >20 years (4)

What type of employer do you have?

- University (1)
- Charitable food relief agency (2)
- Government department – please specify which department and at what level (federal, state/provincial, city/local) (3)
- Not for profit organisation that provides food relief (4)
- Not for profit organisation that does not provide food relief (5)
- Other: please specify (6) ________________________________________________

End of Block: Demographics

Start of Block: Statement of Conflict of Interest

Conflict of Interest In the interests of transparency please state any perceived or actual conflicts of interest you may have. This may be associated with your employment, or could include any funding received from agencies for research or interventions. This may include associations with charitable and corporate food industry, not-for profit organisations and others.

________________________________________________________________

________________________________________________________________

________________________________________________________________

________________________________________________________________

________________________________________________________________

End of Block: Statement of Conflict of Interest

Start of Block: The next questions ask for your views and opinions

Q1 What is your definition of food security?

________________________________________________________________

________________________________________________________________

________________________________________________________________

________________________________________________________________

________________________________________________________________

Q1.1 What other terms do you use and why?

________________________________________________________________

________________________________________________________________

________________________________________________________________

________________________________________________________________

________________________________________________________________

Q2 What is the prevalence of food insecurity in your country? Please give a percentage or number.

________________________________________________________________

________________________________________________________________

________________________________________________________________

________________________________________________________________

________________________________________________________________

Q3 How is household food security measured in your country?  If a specific tool is used please specify

________________________________________________________________

________________________________________________________________

________________________________________________________________

________________________________________________________________

________________________________________________________________

Q3.1 How often is household food security measured in your country?

- Annually (1)
- Every second year (2)
- Every five years (3)
- Ad hoc (4)
- Click to write Choice 6 (6)
- Other (please state) (5) ________________________________________________

Q3.2 What tool **should** be used to measure household food security?

________________________________________________________________

________________________________________________________________

________________________________________________________________

________________________________________________________________

________________________________________________________________

Q3.3 How often do you think household food security **should** be measured?

- Annually (1)
- Every second year (2)
- Every five years (3)
- Ad hoc (4)
- Other (please state) (9) ________________________________________________

Q4 Which sub-populations in your country do you think have been under-represented in these measurements?

________________________________________________________________

________________________________________________________________

________________________________________________________________

________________________________________________________________

________________________________________________________________

Q5 What are the primary determinants of household food insecurity in your country?

________________________________________________________________

________________________________________________________________

________________________________________________________________

________________________________________________________________

________________________________________________________________

Q6 What government initiated strategies are in place in your country to alleviate food insecurity? These could be around national food security or household food security.

________________________________________________________________

________________________________________________________________

________________________________________________________________

________________________________________________________________

________________________________________________________________

Q6.1 Are these government initiated strategies evaluated?

- Yes (1)
- No (2)
- Don't know (3)

Skip To: Q7 If Are these government initiated strategies evaluated? = No

Skip To: Q7 If Are these government initiated strategies evaluated? = Don't know

Q6.2 How are these government initiated strategies evaluated?

________________________________________________________________

________________________________________________________________

________________________________________________________________

________________________________________________________________

________________________________________________________________

Q6.3 Based on you knowledge of the evaluations, how effective are they?

________________________________________________________________

________________________________________________________________

________________________________________________________________

________________________________________________________________

________________________________________________________________

Q7 What community/not for profit sector strategies are in place in your country to alleviate household food insecurity?

________________________________________________________________

________________________________________________________________

________________________________________________________________

________________________________________________________________

________________________________________________________________

Q7.1 How effective are these community not for profit sector strategies?

________________________________________________________________

________________________________________________________________

________________________________________________________________

________________________________________________________________

________________________________________________________________

Q7.2 How are the community, not-for-profit sector strategies funded?

________________________________________________________________

________________________________________________________________

________________________________________________________________

________________________________________________________________

________________________________________________________________

Q7.3 What are the real or perceived conflicts of interest in this funding?

________________________________________________________________

________________________________________________________________

________________________________________________________________

________________________________________________________________

________________________________________________________________

Q8 Does your country have a constitutional guarantee to Right to Food?

- Yes (1)
- No (2)
- Don't know (3)

Display This Question:

If Does your country have a constitutional guarantee to Right to Food? = Yes

Q8.1 How has this been deployed, implemented?

________________________________________________________________

________________________________________________________________

________________________________________________________________

________________________________________________________________

________________________________________________________________

Display This Question:

If Does your country have a constitutional guarantee to Right to Food? = No

Q8.1 Why do you think this is the case?

________________________________________________________________

________________________________________________________________

________________________________________________________________

________________________________________________________________

________________________________________________________________

Q9 Do you think there is political will to improve household food security in your country?

- Yes (1)
- No (2)
- Don't know (3)

Display This Question:

If Do you think there is political will to improve household food security in your country? = Yes

Q9.1 What evidence do you have?

________________________________________________________________

________________________________________________________________

________________________________________________________________

________________________________________________________________

________________________________________________________________

Display This Question:

If Do you think there is political will to improve household food security in your country? = No

Q9.2 Why do you think this is the case?

________________________________________________________________

________________________________________________________________

________________________________________________________________

________________________________________________________________

________________________________________________________________

Q10 Do you think there is public will to improve household food security in your country?

- Yes (1)
- No (2)
- Don't know (3)

Display This Question:

If Do you think there is public will to improve household food security in your country? = Yes

Q10.1 What evidence do you have?

________________________________________________________________

________________________________________________________________

________________________________________________________________

________________________________________________________________

________________________________________________________________

Display This Question:

If Do you think there is public will to improve household food security in your country? = No

Q10.2 Why do you think this is the case?

________________________________________________________________

________________________________________________________________

________________________________________________________________

________________________________________________________________

________________________________________________________________

Q11 What do you think are the primary policy strategies to be implemented to ensure a right to food? You may want to identify specific sub-populations.

________________________________________________________________

________________________________________________________________

________________________________________________________________

________________________________________________________________

________________________________________________________________

Q11.1 What are the barriers to implementing these strategies?

________________________________________________________________

________________________________________________________________

________________________________________________________________

________________________________________________________________

________________________________________________________________

Q12 What other strategies need to be implemented to alleviate household food insecurity (across access, availability, utilisation and stability/sustainability)?

________________________________________________________________

________________________________________________________________

________________________________________________________________

________________________________________________________________

________________________________________________________________

Q12.1 Who should be responsible for these strategies?

________________________________________________________________

________________________________________________________________

________________________________________________________________

________________________________________________________________

________________________________________________________________

Q12.2 How should they be funded?

________________________________________________________________

________________________________________________________________

________________________________________________________________

________________________________________________________________

________________________________________________________________

Q13 Please add any additional comments

________________________________________________________________

________________________________________________________________

________________________________________________________________

________________________________________________________________

________________________________________________________________

Q49 Participate in ongoing consultation:

- I **would like** to participate in any ongoing Delphi process to reach consensus (1)
- I would **not** like to participate in any ongoing Delphi process to reach consensus (2)

Skip To: End of Block If Participate in ongoing consultation: = I would <strong>not </strong>like to participate in any ongoing Delphi process to reach consensus

Q50 My name is:

________________________________________________________________

Q51 My email address is:

________________________________________________________________

End of Block: The next questions ask for your views and opinions

Start of Block: Block 4

THANK YOU FOR COMPLETING THE SURVEY
Questions / further information about the research project If you have any questions or require further information please contact one of the listed researchers:

Danielle Gallegos [danielle.gallegos@qut.edu.au](mailto:danielle.gallegos@qut.edu.au)

Mariana Chilton [mariana.chilton@drexel.edu](mailto:mariana.chilton@drexel.edu)

Sue Booth     [sue.booth@flinders.edu.au](mailto:sue.booth@flinders.edu.au)

Sue Kleve      [suzanne.kleve@monash.edu.au](mailto:suzanne.kleve@monash.edu.au)

Christina Pollard   [c.pollard@curtin.edu.au](mailto:c.pollard@curtin.edu.au)

Concerns / complaints regarding the conduct of the research project QUT is committed to research integrity and the ethical conduct of research projects.  However, if you do have any concerns or complaints about the ethical conduct of the research project you may contact the QUT Research Ethics Advisory Team on +61 7 3138 5123 or email humanethics@qut.edu.au. The QUT Research Ethics Advisory Team is not connected with the research project and can facilitate a resolution to your concern in an impartial manner.

End of Block: Block 4

Household food insecurity in high income countries: Developing a consensus

Start of Block: Description

**Household Food Insecurity in High Income Countries:
 Developing a Consensus**   Household food security is the “physical, social and economic access to sufficient affordable, quality food for an active and healthy life”. Many industrialised countries focus their attention on food production in the face of climate change. While action in this area is vital to ensure the availability of adequate food at a country level, there is a concern that little attention has been given to ‘within country’ food security at the household level. In high income countries, at a population level about 10% of households are food insecure, and 25% of low to middle income households.  This research aims to develop an international consensus on the definition, framing, monitoring and responses to food insecurity in high income countries.  This is the second survey and is based on the responses received from participants in the first survey. In the first survey questions were asked about definitions and strategies.      The second survey continues to build a consensus related to three areas each with related questions. Section 1: Food security definition and dimensions (17 Questions )
Section 2: Measurement  (4 Questions ) 
Section 3: Exploring strategies to address household food insecurity (3 Questions )

You are invited to participate in this research project because you responded to the first round of the Delphi process in the development  of this consensus. 

Instructions for completing this second survey:
   
The survey is primarily qualitative and depending on the depth of your responses will take between about 30 minutes to complete, online. There are some preliminary demographic questions and a conflict of interest statement to complete. These questions have been collated from the first survey you completed in June 2019. Your responses will determine the level of agreement with the statements for building a consensus.    
 
Your participation in this research project is entirely voluntary. If you agree to participate you do not have to complete any question(s) you are uncomfortable answering. Your decision to participate or not participate will in no way impact upon your current or future relationship with QUT, Curtin, Flinders or Monash Universities. If you do agree to participate you cannot withdraw from the research project after submitting your responses as your responses are not-identifiable.      
 
You will be able to review your responses before submitting and save a copy of your responses after submitting the survey.     
 
Expected benefits:  
It is expected that this research project will not directly benefit you. However, it may benefit the development of national and international agendas for the alleviation of household food insecurity. You will be able to use the results from this process for your own organizational advocacy.    
 
Risks:  
There are no risks beyond normal day-to-day living associated with your participation in this research project.    
 
Privacy and Confidentiality:  
All comments and responses are anonymous and will be treated confidentially unless required by law, or regulatory or monitoring bodies, such as the ethics committee.  The names of individual persons are not required in any of the responses. Any data collected as part of this research project will be stored securely as per QUT’s Management of research data policy. Please note that non-identifiable data from this research project may be used as comparative data in future projects or stored on an open access database for secondary analysis.
 
This survey has ethics clearance from the Queensland University of Technology Human Research Ethics Committee Approval Number 1800001126
 
Research Team:
Professor Danielle Gallegos          Queensland University of Technology
 danielle.gallegos@qut.edu.au   
 
Dr Sue Booth                                 Flinders University                                    
sue.booth@flinders.edu.au

 Dr Sue Kleve                                 Monash University                                    
suzanne.kleve@monash.edu.au

 Dr Christina Pollard                       Curtin University                                        
C.Pollard@curtin.edu.au  
 
Please do not hesitate to contact any of the research team members if you have questions.
 

**Consent to Participate**
Answering yes to the consent question means that you consent to participate and your responses even if you only partially complete the survey will be used.  
 
I consent to participate

- Yes (1)
- No (2)

Skip To: End of Survey If Consent to Participate Answering yes to the consent question means that you consent to participat... = No

End of Block: Description

Start of Block: Demograhics

Q1 **In which country do you mainly reside and work?**

- Australia (1)
- New Zealand (4)
- Canada (2)
- England (3)
- Ireland (5)
- Northern Ireland (6)
- Scotland (7)
- United States of America (8)
- Denmark (9)
- France (11)
- Finland (12)
- Spain (13)
- Germany (14)
- Netherlands (15)
- Other: please specify (10) __________________________________________________

Q2 **Please select up to two responses that most represent your qualifications/expertise.**

- Anthropology (1)
- Epidemiology (2)
- Global studies/international development (3)
- Medicine (4)
- Nutrition (5)
- Paediatrics (6)
- Public health (7)
- Sociology (8)
- Other: please specify (9) __________________________________________________

Q3 **How long have you been working in the area of food security?**

- 0-4 years (1)
- 5-10 years (2)
- 11-20 years (3)
- >20 years (4)

Q4 **Where do you work?**

- University (1)
- Charitable food relief agency (2)
- Government department – please specify which department and at what level (federal, state/provincial, city/local) (3)
- Not for profit organisation that provides food relief (4)
- Not for profit organisation that does not provide food relief (5)
- Other: please specify (6) __________________________________________________

End of Block: Demograhics

Start of Block: Conflict of Interest

Q5 **Conflict of Interest:** In the interests of transparency please state any perceived or actual conflicts of interest you may have. This may be associated with your employment, or could include any funding received from agencies for research or interventions. This may include associations with charitable and corporate food industry, not-for profit organisations and others.

________________________________________________________________

________________________________________________________________

________________________________________________________________

________________________________________________________________

________________________________________________________________

End of Block: Conflict of Interest

Start of Block: Block 12

Section 1
**Food security definition and dimensions**
 

________________________________________________________________

End of Block: Block 12

Start of Block: Definition

Q6
**If we proceed with food security as the preferred term there needs to be a clear, agreed definition of food security to progress action within and across nations. After consultation, the proposed definition is based on the Food and Agriculture Organization’s definition but includes additional elements from previous definitions and encompasses how food is sourced.**
 
 The proposed definition is as follows:   “Food security exists when all people, at all times, have**regular and reliable** physical, social and economic access to sufficient safe, nutritious and **culturally relevant**food that meets their dietary needs and food preferences. This is supported by an environment of adequate sanitation, health services and care for an active and healthy life. This includes the assured ability to acquire acceptable foods in socially acceptable ways without resorting to emergency food supplies, scavenging, stealing, and other coping strategies. ”

|  | Strongly agree (1) | Agree (2) | Neither agree nor disagree (3) | Disagree (4) | Strongly disagree (5) | Don't know (6) |
| --- | --- | --- | --- | --- | --- | --- |
| Please rate your level of agreement with this definition. (1) |  |  |  |  |  |  |

Q6.1 **Please state any comments and/ or modifications.**

________________________________________________________________

________________________________________________________________

________________________________________________________________

________________________________________________________________

________________________________________________________________

Q7 This official definition is not one that is typically used for the **general public** there appears to be a need for a simplified version to describe the concept of food security. There were also concerns that the definition does not adequately capture the experiences of **poverty and hunger**.    **Please write your own twenty word definition food security/insecurity that would be understandable to the general public.**

________________________________________________________________

________________________________________________________________

________________________________________________________________

________________________________________________________________

________________________________________________________________

Q8 **The following definitions are proposed as simplified definitions for use with the general public.**

 **Please rank them in order of preference from 1-5 ( 1 being the most appropriate and 5 being the least appropriate for the general public.)**

______ Food poverty is the inability of people to put food on the table that is healthy and affordable without worry, in ways that maintain their dignity. [currently readability Grade 15] (1)

______ Food insecurity is the inability of people to access, adequate, affordable and acceptable food. [currently readability Grade 15] (2)

______ Food insecurity is the uncertainty about the ability to obtain food. It means you have to settle for less food or food of low quality for your family. [current readability Grade 11] (3)

______ Food equity is access to affordable, appropriate and nutritious food at all times for all people regardless of their situation. (current readability Grade 17] (4)

______ Food insecurity is when individuals and families go hungry because they are unable to afford food to meet their needs. [current readability Grade 15] (5)

Q8.1  **Provide comments and/or modifications (if necessary) to your preferred option.**

________________________________________________________________

________________________________________________________________

________________________________________________________________

________________________________________________________________

________________________________________________________________

Q 9 **The definition of food security in high income countries is underpinned by six dimensions outlined by the FAO.**
 
 **1.      Availability** **2.      Access** **3.      Utilisation** **4.      Stability** **5.      Agency** **6.      Sustainability**   **Do you think that these 6 dimensions capture all of the elements of food security in high income countries?**

- Yes, with no changes to the definition (1)
- No, this does not capture my understanding of this dimension (2)

Skip To: Q 9.1 If The definition of food security in high income countries is underpinned by six dimensions outline... = No, this does not capture my understanding of this dimension

Q 9.1 **Please describe what you think is currently missing in the dimensions of food insecurity for high income countries that is currently not addressed or is unclear.**

________________________________________________________________

________________________________________________________________

________________________________________________________________

________________________________________________________________

________________________________________________________________

End of Block: Definition

Start of Block: Availability Dimension

**Based on the first Delphi the following amendments and clarifications are suggested for defining each of the dimensions.**

 Q 10 
Dimension - Availability 
**Healthy nutritious foods free from adverse substances need to be available at the national, community and local level. Sufficient food variety needs to be available  to enable individuals to choose foods that meet their physical, social and cultural needs need**s.

|  | Strongly agree (1) | Agree (2) | Neither agree or disagree (3) | Disagree (4) | Strongly disagree (5) | Don't know (6) |
| --- | --- | --- | --- | --- | --- | --- |
| Please rate your level of agreement with this definition (1) |  |  |  |  |  |  |

Q10.1 **Please state any comments and/ or modifications.**

________________________________________________________________

________________________________________________________________

________________________________________________________________

________________________________________________________________

________________________________________________________________

End of Block: Availability Dimension

Start of Block: Access Dimension

Q11
**Dimension - Access**
**Access is the other dimension that includes economic, social and physical access. We are proposing that access be divided into three sub-domains.**
 
**Do you agree that access should be divided into economic, social and physical?**

|  | Strongly agree (1) | Agree (2) | Neither agree nor disagree (3) | Disagree (4) | Strongly disagree (5) | Don't know (6) |
| --- | --- | --- | --- | --- | --- | --- |
| Please rate your level of agreement with this definition (1) |  |  |  |  |  |  |

Q11.1 **Please state any comments and/ or modifications.**

________________________________________________________________

________________________________________________________________

________________________________________________________________

________________________________________________________________

________________________________________________________________

Q12 **Economic access** **Individuals, households and communities need sufficient income to meet the needs of daily living without stress, including housing, water, utilities, communication and food. Healthy food should be affordable.**

|  | Strongly agree (1) | Agree (2) | Neither agree nor disagree (3) | Disagree (4) | Strongly disagree (5) | Don't know (6) |
| --- | --- | --- | --- | --- | --- | --- |
| Please rate your level of agreement with this definition (1) |  |  |  |  |  |  |

Q12.1 **Please state any comments and/ or modifications.**

________________________________________________________________

________________________________________________________________

________________________________________________________________

________________________________________________________________

________________________________________________________________

Q 13 **Social access** **All members of communities have access to food irrespective of their circumstances, gender, or age. Food can be acquired in ways that are socially acceptable and just.**

|  | Strongly agree (1) | Agree (2) | Neither agree nor disagree (3) | Disagree (4) | Strongly disagree (5) | Don't know (6) |
| --- | --- | --- | --- | --- | --- | --- |
| Please rate your level of agreement with this definition (1) |  |  |  |  |  |  |

Q13.1 **Please state any comments and/ or modifications.**

________________________________________________________________

________________________________________________________________

________________________________________________________________

________________________________________________________________

________________________________________________________________

Q 14 **Physical access** **Food needs to be accessible where people live. Individuals, households and communities should have access to infrastructure and strategies that optimises access to food without undue burden on household resources.**

|  | Strongly agree (1) | Agree (2) | Neither agree nor disagree (3) | Disagree (4) | Strongly disagree (5) | Don't know (6) |
| --- | --- | --- | --- | --- | --- | --- |
| Please rate your level of agreement with this definition (1) |  |  |  |  |  |  |

Q14.1 **Please state any comments and/ or modifications.**

________________________________________________________________

________________________________________________________________

________________________________________________________________

________________________________________________________________

________________________________________________________________

End of Block: Access Dimension

Start of Block: Utilisation Dimension

Q 15 **Dimension - Utilisation** **We are proposing that the utilisation dimension be divided into four sub-domains:** **Access to household equipment** **Food literacy** **Time** **Water, sanitation and health (WaSH)**
 
**Do you agree that access should be divided into four sub-domains?**

|  | Strongly agree (1) | Agree (2) | Neither agree nor disagree (3) | Disagree (4) | Strongly disagree (5) | Don't know (6) |
| --- | --- | --- | --- | --- | --- | --- |
| Please rate your level of agreement with this definition (1) |  |  |  |  |  |  |

Q 15.1 **Please state any comments and/ or modifications.**

________________________________________________________________

________________________________________________________________

________________________________________________________________

________________________________________________________________

________________________________________________________________

Q 16 **Access to household equipment** **Individuals and households require access to equipment that is in good working order that enables them to convert food into meals that meet their health, social and cultural needs.**

|  | Strongly agree (1) | Agree (2) | Neither agree nor disagree (3) | Disagree (4) | Strongly disagree (5) | Don't know (6) |
| --- | --- | --- | --- | --- | --- | --- |
| Please rate your level of agreement with this definition (1) |  |  |  |  |  |  |

Q 16.1 **Please state any comments and/ or modifications.**

________________________________________________________________

________________________________________________________________

________________________________________________________________

________________________________________________________________

________________________________________________________________

Q 17 **Food Literacy** **Communities, households and individuals need the knowledge, skills and behaviours that empower them to plan, manage, select, prepare and eat foods that meet their health, social and cultural needs.**

|  | Strongly agree (1) | Agree (2) | Neither agree nor disagree (3) | Disagree (4) | Strongly disagree (5) | Don't know (6) |
| --- | --- | --- | --- | --- | --- | --- |
| Please rate your level of agreement with this definition (1) |  |  |  |  |  |  |

Q17.1 **Please state any comments and/ or modifications.**

________________________________________________________________

________________________________________________________________

________________________________________________________________

________________________________________________________________

________________________________________________________________

Q 18 **Time** **Time pressures/demands of individuals or households that impact on food provisioning (e.g to plan, manage, select and prepare food). The relationship between the time available (work, parenting and other demands) versus the perceived time deemed to undertake food provisioning tasks.**

|  | Strongly agree (1) | Agree (2) | Neither agree nor disagree (3) | Disagree (4) | Strongly disagree (5) | Don't know (6) |
| --- | --- | --- | --- | --- | --- | --- |
| Please rate your level of agreement with this definition (1) |  |  |  |  |  |  |

Q 18.1 **Please state any comments and/ or modifications.**

________________________________________________________________

________________________________________________________________

________________________________________________________________

________________________________________________________________

________________________________________________________________

Q 19 **Water, sanitation and health (WaSH)**
 **All communities, households and individuals need access to a water supply that is safe and affordable, a sanitation system that prevents disease and the knowledge and skills to keep the water and food supply safe to consume.**

|  | Strongly agree (1) | Agree (2) | Neither agree nor disagree (3) | Disagree (4) | Strongly disagree (5) | Don't know (6) |
| --- | --- | --- | --- | --- | --- | --- |
| Please rate your level of agreement with this definition (1) |  |  |  |  |  |  |

Q 19.1 **Please state any comments and/ or modifications.**

________________________________________________________________

________________________________________________________________

________________________________________________________________

________________________________________________________________

________________________________________________________________

End of Block: Utilisation Dimension

Start of Block: Stability Dimension

Q 20 **Dimension - Stability** **All communities, households and individuals can ensure the availability of, access to and utilisation of healthy, nutritious food in the event of shocks that may occur at a global, national, community or household level. This would include climate change, economic crises, pandemics, violence (conflict as well as personal violence and trauma) and cyclical events (e.g weather).**

|  | Strongly agree (1) | Agree (2) | Neither agree nor disagree (3) | Disagree (4) | Strongly disagree (5) | Don't know (6) |
| --- | --- | --- | --- | --- | --- | --- |
| Please rate your level of agreement with this definition (1) |  |  |  |  |  |  |

Q 20.1 **Please state any comments and/ or modifications.**

________________________________________________________________

________________________________________________________________

________________________________________________________________

________________________________________________________________

________________________________________________________________

End of Block: Stability Dimension

Start of Block: New Dimensions

**In 2020, the FAO identified an additional two dimensions to the food security framework. These are agency and sustainability.**

Q 21 **Dimension - Agency:**
 **The** [FAO](http://www.fao.org/3/ca9731en/ca9731en.pdf)**(2020) define this as:** **“Individuals or groups having the capacity to act independently to make choices about what they eat, the foods they produce, how that food is produced, processed, and distributed, and to engage in policy processes that shape food systems. The protection of agency requires socio-political systems that uphold governance structures that enable the achievement of Food and Nutrition Security for all.”**
 **This definition encompasses both food sovereignty and the right to food governance.**

|  | Strongly agree (1) | Agree (2) | Neither agree nor disagree (3) | Disagree (4) | Strongly disagree (5) | Don't know (6) |
| --- | --- | --- | --- | --- | --- | --- |
| Please rate your level of agreement with this definition (1) |  |  |  |  |  |  |

Q21.1 **Please state any comments and/ or modifications.**

________________________________________________________________

________________________________________________________________

________________________________________________________________

________________________________________________________________

________________________________________________________________

Q 22 **Dimension - Sustainability

 The**[FAO (2020)](http://www.fao.org/3/ca9731en/ca9731en.pdf)**define this dimension as:
 “Food system practices that contribute to long-term regeneration of natural, social and economic systems, ensuring the food needs of the present generations are met without compromising the food needs of future generations.”

 This definition encompasses both food sovereignty and the right to food governance.**

|  | Strongly agree (1) | Agree (2) | Neither agree nor disagree (3) | Disagree (4) | Strongly disagree (5) | Don't know (6) |
| --- | --- | --- | --- | --- | --- | --- |
| Please rate your level of agreement to the definition as provided. (1) |  |  |  |  |  |  |

Q 22.1 **Please state any comments and/ or modifications.**

________________________________________________________________

________________________________________________________________

________________________________________________________________

________________________________________________________________

________________________________________________________________

End of Block: New Dimensions

Start of Block: 2. Measurement

Section 2
**Measurement**
**A range of measurement instruments are used in high income countries to identify household food security at the population level.**
**Please rate your agreement with the following statements**

Q 23 **Essential components of the measurement instrument.**
**The measurement instrument should be able to:**

|  | Strongly Agree (1) | Agree (2) | Neither Agree or Disagree (3) | Disagree (4) | Strongly disagree (5) | Don't know (6) |
| --- | --- | --- | --- | --- | --- | --- |
| Assess the level of severity (4) |  |  |  |  |  |  |
| Capture whether a household is worrying about food (5) |  |  |  |  |  |  |
| Detect hunger (6) |  |  |  |  |  |  |
| Be applied at a household or individual level (7) |  |  |  |  |  |  |
| Indicate the impact of food insecurity on children (8) |  |  |  |  |  |  |

Q 24 **Implementation of the measurement instrument should be:**

|  | Strongly Agree (1) | Agree (2) | Neither Agree or Disagree (3) | Disagree (4) | Strongly Disagree (5) | Don't know (7) |
| --- | --- | --- | --- | --- | --- | --- |
| Government mandated (1) |  |  |  |  |  |  |
| Population-based surveillance (2) |  |  |  |  |  |  |
| Able to be administered in a variety of forms (interviewer-administered; self-complete) (3) |  |  |  |  |  |  |
| Still valid to use over different time periods: for example over the last 12 months, over the last three months, over the last month (4) |  |  |  |  |  |  |
| Allow for comparison across time-points (temporality) (5) |  |  |  |  |  |  |
| Reported annually (8) |  |  |  |  |  |  |
| Implemented in a way that ensures all potential populations experiencing food insecurity are captured e.g those without a telephone, those living in caravan/trailer parks, those without a fixed address (9) |  |  |  |  |  |  |

Q23.1 **Please comment on any of the above**

________________________________________________________________

________________________________________________________________

________________________________________________________________

________________________________________________________________

________________________________________________________________

Q 24  **The measurement tools that are currently in use internationally are listed below.**
 
**Please rank in order of preference (1-4,where 1 is your most preferred and 4 is your least preferred) for the tool you think would be most useful in meeting your respective country’s policy needs.**
 
**The link beside each question will take you to the full questionnaire.**

______ USDA Household Food Security Survey Module - This is a comprehensive tool that identifies levels of severity of food insecurity as well as the impact on children. There is a short-form of six questions, a 10 item version for adult only households and an 18 item version for households with children. Time period is usually the last 12 months. https://www.ers.usda.gov/media/8271/hh2012.pdf (1)

______ Household Food Insecurity Access Scale - This scale has been developed by FANTA and is derived from the USDA HFSSM and informs the FAO FIES tool. There is a generic form which has nine questions. Impact on children is not assessed. Time period is the last month. https://www.fantaproject.org/sites/default/files/resources/HFIAS_ENG_v3_Aug07.pdf (11)

______ FAO Food Insecurity Experience Scale - This is an eight item scale that can be analysed statistically to determine severity. It is unable to discern the impact of food insecurity for children. Time period is usually the last 12 months.http://www.fao.org/policy-support/tools-and-publications/resources-details/en/c/1236494/ (2)

______ Food Insufficiency Question - This is one question that asks about running out of money for food. Time period is usually the last 12 months. (7)

Q 25  **Is there another tool that should be considered?
 If yes please indicate the tool name.**

- Yes (4) __________________________________________________
- No (5)

End of Block: 2. Measurement

Start of Block: 3. Exploring strategies to address household food insecurity

 Section 3 Exploring strategies to address household food insecurity   There are a range of current and potential actions/strategies to address household food insecurity and its determinants. These may vary within and across countries and operate at local or national levels.      .

| 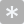 |
| --- |

Q 26  **National level primary policy/social protection strategies**   **Below is a  list of government primary policy/social protection strategies that have been implemented in high income countries that were identified from the first round. Many of these come under the umbrella of social protection.** **You have been asked to provide your government with what you believe to be the three most effective strategies for alleviating food insecurity.**   **Please number your top three in order of priority from 1-3, with 1 being your highest priority.**

______ Social welfare payments (social security benefits, unemployment benefits, single parent support, housing assistance) (4)

______ Universal basic income (7)

______ Legislated minimum wage - Minimum wage is a living wage (8)

______ Comprehensive poverty alleviation strategy (10)

______ Work ready training programs (11)

______ Employment & working conditions: Livelihood and employment generation &promotion for growth/scalability (12)

______ Employment & working conditions: actions to address underemployment/insecure employment conditions(including policy) (13)

______ Subsidised utilities (gas/electricity/water) for low income earners (14)

______ Income management policies e.g cashless debit card for Indigenous communities (15)

______ Measurement, mapping and reporting on food insecurity (17)

______ Social/Public Housing support services and Policy (18)

______ Violence support services and policy (19)

______ Actions to support access to education (20)

______ Strategies to support racism reduction (21)

______ Overarching multi-sector food and nutrition security policy at national level (23)

______ Program for child poverty reduction and wellbeing legislation (24)

______ Government funded food security programs e.g SNAP, WIC (25)

Q 26.1 **Please provide any comments on the rationale of your choice 1-3. For example these may be currently implemented in your country but criteria for eligibility may not be equitable for example the social welfare payment may be under the poverty line, there may be onerous conditions for eligibility.**

________________________________________________________________

________________________________________________________________

________________________________________________________________

________________________________________________________________

________________________________________________________________

Q26.2 **If there is a strategy not currently listed and you believe it to be more effective, please identify this in the comment box.**

________________________________________________________________

________________________________________________________________

________________________________________________________________

________________________________________________________________

________________________________________________________________

| 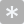 |
| --- |

Q 27 **National level**   **Imagine you have been approached by government to identify three priority strategies for investment at the national level that will sustainably alleviate food insecurity within communities.** **Please number your top three in order of priority from 1-3, with 1 being your highest priority. For the strategies you select a comment box will appear, in your comments provide a rationale for your choice**

______ Mapping and evaluating national and local programs (4)

______ Universal free school meals (breakfast, lunch, snacks) (6)

______ Means tested school meals (7)

______ Food literacy programs in schools (9)

______ Food rescue programs (11)

______ Resilience and agility of food supply chains and environments (12)

______ Retail food environment -supermarkets -policy pricing strategy (13)

______ Subsidies on healthy food choices (14)

______ Taxes on unhealthy food choices (15)

Q 27.1 **Please provide any comments on the rationale of your choice 1-3.**

________________________________________________________________

Q 27.2 **If there is a strategy not currently listed and you believe it to be more effective please identify this in the comment box.**

________________________________________________________________

________________________________________________________________

________________________________________________________________

________________________________________________________________

________________________________________________________________

| 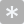 |
| --- |

Q28 **Community/local level** **Imagine you have been approached by government to identify three priority strategies for investment at the local or community level that will sustainably alleviate food insecurity within communities.** **Please number your top three in order of priority from 1-3, with 1 being your highest priority. For the strategies you select a comment box will appear, in your comments provide a rationale for your choice.**

______ Social supermarkets or social solidarity stores (1)

______ Emergency food relief, food assistance programs (4)

______ Social cafes (5)

______ Cooking or other food literacy programs (6)

______ Budgeting programs (7)

______ Food waste reduction campaign (9)

______ Access to safe drinking water (10)

______ Community programs that provide a suite of services e.g. food access, financial access, social inclusion growing food, provides food relief, financial and housing advice, counselling, links to training and education, employment opportunities (12)

______ Food Hubs (14)

______ Community gardens (15)

______ Community kitchens (16)

______ Farmers markets and community food markets (17)

______ Localised food production (18)

______ Meal provisioning for community dwelling elderly e.g Meals on Wheels, Congregate (meals provided for the elderly at a venue) (19)

Q 28.1 **Please provide any comments on the rationale of your choice 1-3.**

________________________________________________________________

Q 28.2 **If there is a strategy not currently listed and you believe it to be more effective please identify this in the comment box.**

________________________________________________________________

________________________________________________________________

________________________________________________________________

________________________________________________________________

________________________________________________________________

End of Block: 3. Exploring strategies to address household food insecurity

Start of Block: Additional comments

Section 4 **Additional Comments**   **Please feel free to make any additional comments**

________________________________________________________________

________________________________________________________________

________________________________________________________________

________________________________________________________________

________________________________________________________________

End of Block: Additional comments
